# Identification of Nonfunctional Alternatively Spliced Isoforms of STING in Human Acute Myeloid Leukemia

**DOI:** 10.1158/2767-9764.CRC-24-0095

**Published:** 2024-03-25

**Authors:** Akash R. Boda, Arthur J. Liu, Susana Castro-Pando, Benjamin T. Whitfield, Jeffrey J. Molldrem, Gheath Al-Atrash, Maria Emilia Di Francesco, Philip Jones, Casey R. Ager, Michael A. Curran

**Affiliations:** 1 Immunology Program, The University of Texas MD Anderson UTHealth Graduate School of Biomedical Sciences, Houston, Texas.; 2 Department of Immunology, The University of Texas MD Anderson Cancer Center, Houston, Texas.; 3 Department of Gastrointestinal Medical Oncology, The University of Texas MD Anderson Cancer Center, Houston, Texas.; 4 Department of Stem Cell Transplantation and Cellular Therapy, The University of Texas MD Anderson Cancer Center, Houston, Texas.; 5 Institute for Applied Cancer Science, The University of Texas MD Anderson Cancer Center, Houston, Texas.; 6 Department of Immunology, The Mayo Clinic, Scottsdale, Arizona.

## Abstract

Lack of robust activation of Stimulator of Interferon Genes (STING) pathway and subsequent induction of type I IFN responses is considered a barrier to antitumor immunity in acute myeloid leukemia (AML). Using common human AML cell lines as *in vitro* tools to evaluate the efficacy of novel STING agonists, we found most AML lines to be poor producers of IFNs upon exposure to extremely potent agonists, suggesting cell-intrinsic suppression of STING signaling may occur. We observed unexpected patterns of response that did not correlate with levels of STING pathway components or of known enzymes associated with resistance. To identify a genetic basis for these observations, we cloned and sequenced *STING* from the cDNA of human AML cell lines and found both frequent mutations and deviations from normal RNA splicing. We identified two novel spliced isoforms of *STING* in these lines and validated their expression in primary human AML samples. When transduced into reporter cells, these novel STING isoforms exhibited complete insensitivity to agonist stimulation. These observations identify alternative splicing as a mechanism of STING pathway suppression and suggest that most AML silences the STING pathway through direct modification rather than through engagement of external inhibitory factors.

**Significance:**

We find that AML acquires resistance to innate immune activation via the STING pathway through aberrant splicing of the STING transcript including two novel forms described herein that act as dominant negatives. These data broaden understanding of how cancers evolve STING resistance, and suggest that the AML tumor microenvironment, not the cancer cell, should be the target of therapeutic interventions to activate STING.

## Introduction

Robust regulation of the cGAS-STING cytosolic nucleic acid detection pathway is critical for preventing inflammatory pathology following infection or DNA damage but can be leveraged to promote immune hyporesponsiveness in cancer. STING is an endoplasmic reticulum-resident pattern recognition receptor that binds cyclic dinucleotide (CDN) molecules derived from bacteria or the upstream cytoplasmic DNA sensor cGAS during viral infection, necrosis, or genotoxic stress (1, 2). Upon CDN binding, STING activates TBK1 to engage both IRF3 and NFκB signaling pathways, whose concerted actions induce release of IFNβ, among other proinflammatory factors (3, 4). STING-mediated IFNβ release is considered critical for innate activation and priming of adaptive immunity against cancer (5, 6), thus suppression of STING signaling is a logical mechanism of tumor immune evasion (7–9). Like other innate immune receptors, STING can be regulated at genetic (10), epigenetic (9), transcriptional (11, 12), and posttranslational levels (13, 14); however, to date only epigenetic silencing has been described in cancer (7–9). We sought to further understand the mechanisms by which tumors suppress STING activation to identify tumors susceptible to CDNs and to inform design of novel agonists capable of circumventing these adaptations.

Poor induction of IFNβ is a proposed means of immune evasion in acute myeloid leukemia (AML), suggesting STING signaling may be a rate-limiting factor in immune control of this disease (15). To therapeutically drive STING signaling in this setting, we developed novel STING agonists and demonstrated that their potency, in some cases, exceeds that of any cGAMP-analog CDN compound described previously. Using these novel agonists, we sought to determine the extent to which the IFN response of human AML lines was limited by the potency of the STING agonist versus by intrinsic resistance mechanisms. Initially, we confirmed that common human AML lines are poor producers of IFNs upon exposure to CDNs, which could not be readily explained by expression levels of STING or cGAS. We also did not observe consistent upregulation of potential regulators of CDN availability including ectonucleotide pyrophosphatase/phosphodiesterase 1 (ENPP1; ref. 16) or three prime repair exonuclease 1 and 2 (TREX1, TREX2; refs. 17–19). Thus, we sought to identify a genetic basis for these observations by cloning and sequencing *STING* from the cDNA of each AML line. This analysis revealed multiple instances of a novel putative splice variant which retains intron 3 of *STING* and causes premature termination of translation. We not only confirmed the presence of this novel isoform in a high frequency of primary AML bone marrow aspirates, but also discovered an additional splice variant lacking *STING* exon 4. Functional assays indicate these novel variants are unresponsive to all CDNs tested. Together, these studies identify two novel splice variants of STING and suggest that expression of these nonfunctional variants may contribute to suppression of STING-mediated IFNβ production in AML.

## Materials and Methods

### Cells

Human AML cell lines were purchased from ATCC (Kasumi-1, Mv-411, AML193, HL-60) or DSMZ (U937, OCI-AML3) all in 2017–2018. THP-1 and HEK-Blue-ISG-KO-STING were purchased from Invivogen in 2017–2018. Upon receipt, cells were expanded to make original cryopreserved stocks with each subsequent experiment using a new original stock. Human AML cell lines were cultured either in complete RPMI (10% FBS, 100 U/mL penicillin, 100 mg/mL streptomycin sulfate, and 2 mmol/L l-glutamine) or as described in suppliers’ guidelines. THP-1 and HEK-Blue-ISG-KO-STING cells were cultured according to manufacturer's instructions (Invivogen). All cell lines were monitored for cell line authenticity at the MD Anderson Cancer Center in-house core using the Cytogenetics and short tandem repeat sequencing to assess for characteristics chromosomal/mutational status of respective cells lines. Cell lines were routinely tested for *Mycoplasma* at the MD Anderson Cancer Center in-house core using Lonza MycoAlert Kit. Cryopreserved primary bone marrow aspirates from patients with AML treated at MD Anderson Cancer Center were received as a gift from Drs. Gheath Al-Atrash and Jeffrey Molldrem.

### ELISA and Reporter Assays

AML cell lines were plated 1 × 10^5^ cells/well in flat-bottom 96-well plates, then stimulated with 10 µg indicated CDN for 72 hours. Fresh supernatants were utilized for IFNβ (PBL Assay Science) or IFNα (eBioscience) ELISAs according to manufacturer's protocols. HEK-Blue-ISG-KO-STING cells were stimulated according to manufacturer's instructions (Invivogen). Stable transduction of STING alleles into HEK-Blue cells by retroviral transduction was conducted according to manufacturer's protocols for wild-type (WT), HAQ, and R232H alleles (pUNO1 vectors; Invivogen). STING-I3R and STING-Ex4D alleles were cloned into pMG-Lyt2 retroviral vectors and transduced into HEK-Blue-ISG-KO-STING or HEK-Blue-STING WT cells as described previously (20).

### STING Cloning

Total RNA was isolated from ≤5 × 10^6^ cells of indicated source using the RNeasy Mini Kit (Qiagen). Total RNA was converted to cDNA using SuperScript IV Reverse Transcriptase (Thermo Fisher Scientific). *STING* was PCR amplified (using primers: 5′-TGCGGCCGCAGAAGATGCCCCACTCCAGCCTGCAT-3′ forward, 5′-GTCGACTGGGTCTCAAGAGAAATCCG-3′ reverse), gel-extracted (Thermo Fisher Scientific GeneJET Gel Extraction Kit), and cloned into the pCR-Blunt vector for sequencing using the ZeroBlunt PCR Cloning Kit (Invitrogen).

### Quantitative PCR

Gene expression analysis was conducted using TaqMan Fast Advanced Master Mix (Applied Biosystems) and was analyzed on a ViiA 7 Real-Time PCR System (Thermo Fisher Scientific). The following TaqMan probes were purchased from Thermo Fisher Scientific: *STING*/TMEM173 (Hs00736955_g1), *cGAS*/MB21D1 (Hs00403553_m1), *TREX1* (Hs03989617_s1), *TREX2* (Hs00273080_s1), *ENPP1* (Hs01054048_m1), and *GAPDH* (Hs02786624_g1).

### Western Blotting

STING WT allele with His6x tag and HA tag, and STING-I3R and STING-Ex4D alleles with His6x tag were cloned into pGC-YFP retroviral vectors followed by transduction into HEK-Blue-ISG-KO-STING cells (Invivogen). Cells were sorted and YFP expression was assessed by flow cytometry. Cells were washed with cold PBS and lysed with RIPA Cell Lysis Buffer (Thermo Fisher Scientific, 89901) containing protease and phosphatase inhibitors. Following cell lysate protein quantitation with Pierce bicinchoninic acid Protein Assay Kit (Thermo Fisher Scientific, 23225), equivalent amounts of protein were loaded onto SDS-PAGE gels for electrophoresis (4% to 15% gel). Proteins were transferred onto nitrocellulose membranes and blocked in 5% BSA for 1 hour. The membranes were incubated with anti-His6x antibody (Invitrogen, MA1-21315) at 1:1,000 overnight at 4°C and then with horseradish peroxidase (HRP)-conjugated secondary antibody (Cell Signaling Technology, 7076P2) at 1:4,000 for 1.5 hours at room temperature. β-actin loading controls were incubated with mouse anti-human β-actin-HRP (Abcam, ab20272) at 1:3,000 for 30 minutes. The signal was detected by chemiluminescence.

### Data Availability

Data were generated by the authors and included in the article. Raw data supporting the findings in this study can be obtained from the author upon reasonable request.

## Results

### Evaluating Activity of Novel STING Agonists on Alternate STING Isoforms

**FIGURE 1:**
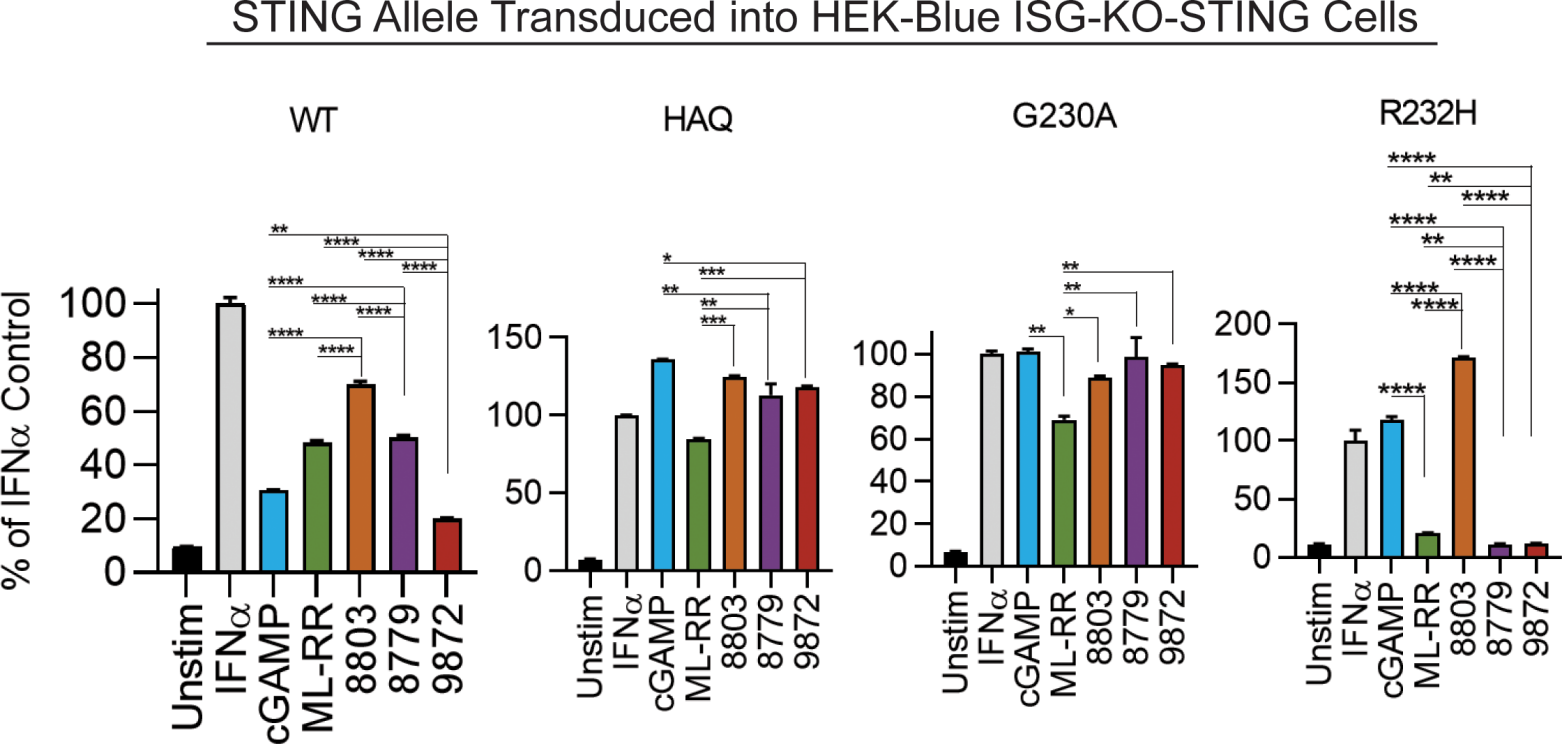
Evaluating activity of novel STING agonists on alternate STING isoforms. A total of 5 × 10^4^ HEK-Blue reporter cells expressing indicated STING alleles were incubated with 100 µg/mL CDN for 24 hours. IRF3/9 activity was measured by secreted alkaline phosphatase (SEAP) assay. IFNα positive control indicates presence and functionality of the IRF3/9 SEAP reporter in these cells. Statistical significance was calculated using repeated measures one-way ANOVA (*n* = 2). ns, not significant; *, *P* < 0.05; **, *P* < 0.01; ***, *P* < 0.001; ****, *P* < 0.0001.

We previously designed and synthesized several novel STING agonists with modifications to the cyclic di-AMP dinucleotide, and evaluated their relative activity compared with known CDNs using established *in vitro* and *in vivo* systems. These studies identified two agonists (8779 and 8803) which demonstrate equivalent or superior potency relative to the clinical compound ML-RR-S2-CDA (ML-RR) in activating WT STING *in vitro* and *in vivo* (21). To determine whether these CDNs can stimulate alternative STING isoforms, we treated HEK-Blue reporter cells reconstituted with either WT, HAQ, G230A, or R232H STING alleles (Fig. 1). We found 8803 and 8779 sufficiently activate WT, HAQ, and G230A alleles. Interestingly, only 8803 and 2′3′-cGAMP elicited detectable activation of R232H. To determine whether the efficacy of 8803 is STING dependent, we stimulated WT and STING-deficient murine splenocytes with CDNs and performed ELISA for IFNβ (Supplementary Fig. S1A). We next performed *in vivo* dose titrations to describe the bioactive doses of these CDNs in a bilateral B16-OVA model and found that systemic immunity equivalent to that elicited by intratumoral ML-RR could be achieved with 1/10th the dose of 8803, with local efficacy observed at doses as low as 10 ng (21). Analogous evaluation of 8779 demonstrated equivalent therapeutic activity relative to ML-RR (Supplementary Fig. S1B). These experiments validated 8779 and 8803 as potent novel CDNs with activity against known germline-encoded STING isoforms and therapeutic efficacy in a standard model of murine cancer.

### Common AML Lines Exhibit Variable Responses to Novel STING Agonists *In Vitro*

**FIGURE 2:**
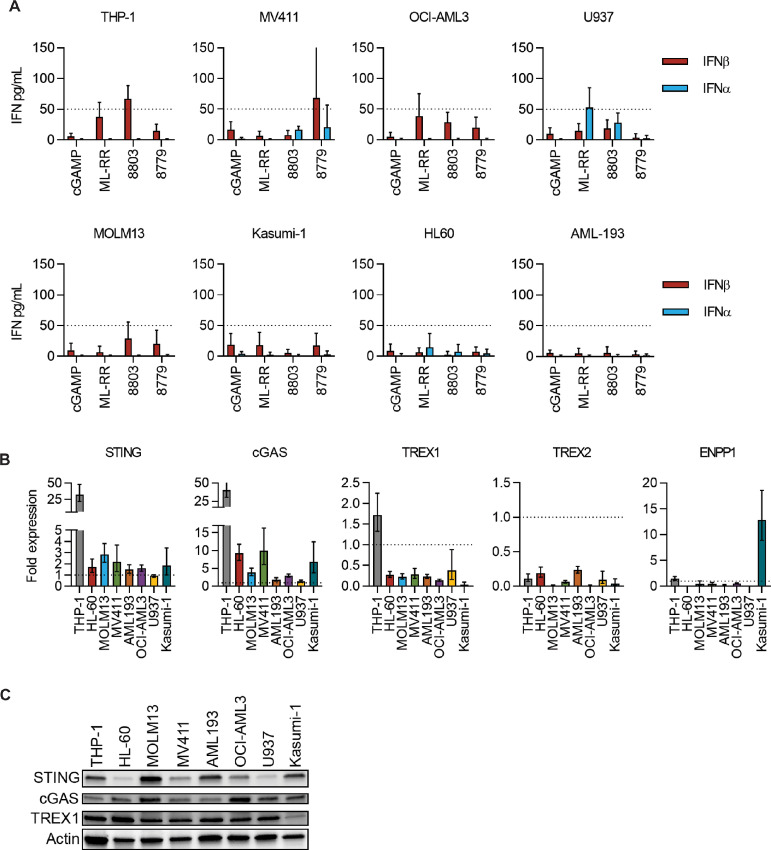
Evaluation of the STING pathway across common AML cell lines. **A,** Indicated AML cell lines plated 1 × 10^5^ cells/well were stimulated 72 hours with CDNs at 10 µg/mL, and supernatants were harvested for ELISA (*n* = 5). An assay detection limit of 50 pg/mL is shown. qPCR analysis (**B**) and immunoblot (**C**) of STING pathway and resistance genes across AML lines (*n* = 1). For qPCR, data represent GAPDH-normalized fold expression relative to normal CD11b^+^ PBMC controls.

We next sought to determine whether our novel CDNs can elicit IFNα/β production in AML cells. We thus performed ELISA for IFNα/β on supernatants from eight human AML cell lines following CDN stimulation. Overall, only THP-1 and MV411 consistently exhibited IFNβ release at levels that surpassed the detection threshold of our assays, while IFNα was largely undetectable in all cell lines except for U937 (Fig. 2A). Interestingly, while THP-1 and MV411 could produce detectable IFNβ, they did so in response to different agonists—8803 and 8779, respectively (Fig. 2A). To better understand this disparate response, we investigated potential mechanisms by which AML cells regulate STING signaling. First, we used qPCR to identify baseline transcript levels of *STING* and *cGAS* and found no evidence of downregulation relative to healthy donor CD11b^+^ peripheral blood mononuclear cells (PBMC; Fig. 2B). Of note, THP-1 cells possess 30- to 40-fold more *STING* and *cGAS* transcript relative to other AML cell lines, while STING protein appeared diminished in HL-60, MV411, OCI-AML3, and U937 (Fig. 2C). In addition, we investigated expression of exogenous regulators of CDN availability including DNA exonucleases *TREX1* and *TREX2* and the CDN phosphodiesterase *ENPP1*. Only THP-1 expressed *TREX1* at levels similar to CD11b^+^ PBMC controls; however, TREX1 protein appeared comparable across cell lines except for the reduced levels observed in Kasumi-1. None of the lines expressed significant transcript levels of *TREX2*. While Kasumi-1 exhibited elevated levels of *ENPP1* transcript, amplification of this gene occurred near the detection limit of qPCR, suggesting this gene is expressed at extremely low levels. Together, these data indicate that altered expression of cGAS-STING pathway components or exogenous regulators of CDN availability are not probable mechanisms of STING pathway suppression in AML.

### Cloning STING Reveals Novel Alternatively Spliced Isoforms in Human AML

**FIGURE 3:**
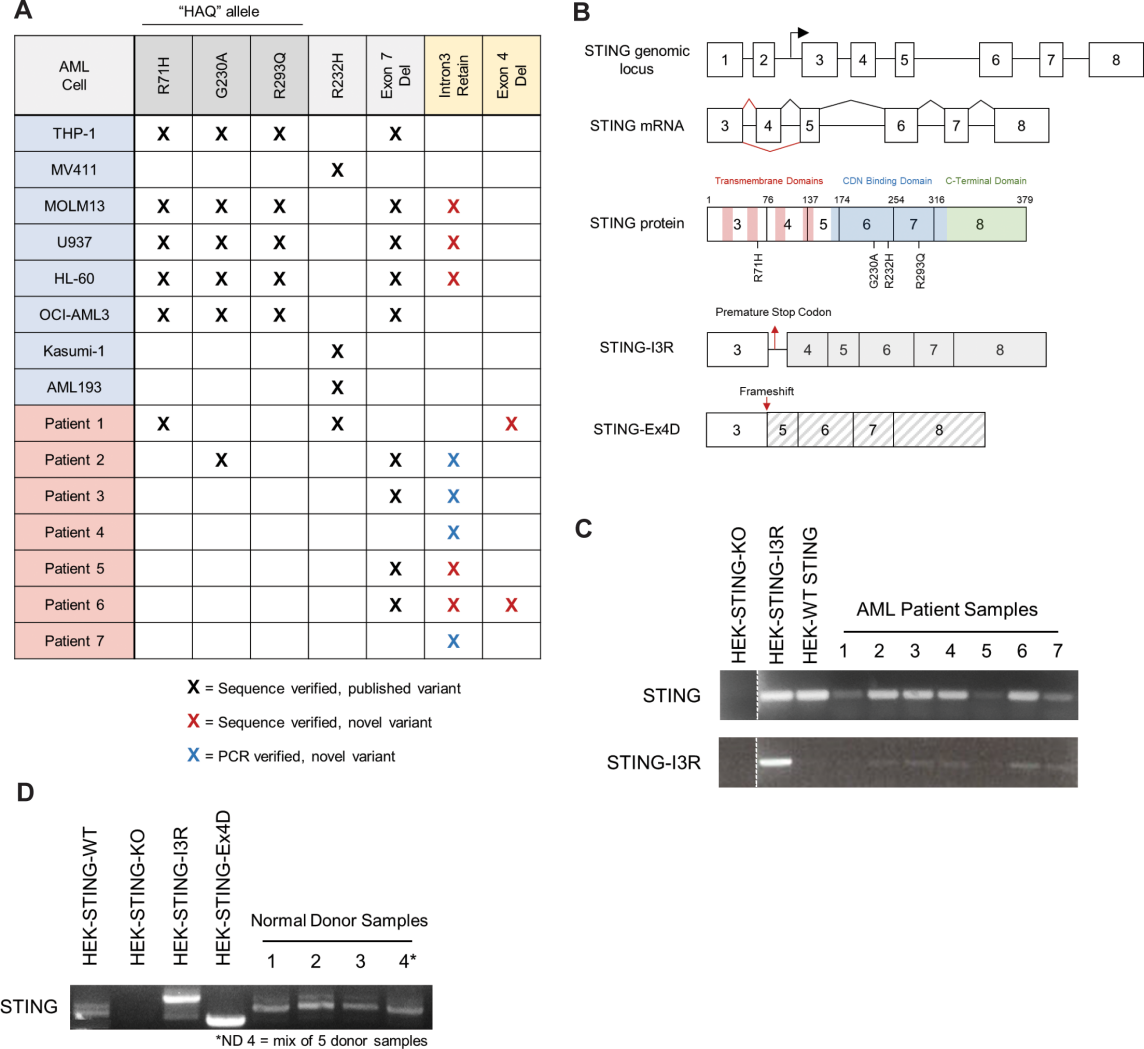
Identification of STING germline and transcript variants in AML. **A,** Summary of sequencing results indicating identification of known STING variant alleles and novel spliced isoforms. **B,** Structure of STING genomic locus and annotated coding sequence maps for WT, STING-I3R, and STING-Ex4D. Identification and validation of STING-I3R allele in primary bone marrow aspirates (**C**) and in normal donors (**D**) using primers to selectively amplify WT or I3R STING alleles.

An alternative hypothesis for the observed CDN insensitivity in AML is selective expression of dysfunctional germline or transcript variants of *STING*. To investigate this hypothesis, we cloned and sequenced *STING* from cDNA of each cell line. Using this approach, we observed the full spectrum of known germline *STING* variants at high frequency, including HAQ and R232H alleles and the previously described MRP splice variant (Fig. 3A and B). Remarkably, we found that none of the AML cell lines were homozygous for WT *STING*. In MOLM13, U937, and HL-60, we discovered a previously unidentified splice variant of *STING* which retains intron 3 in the coding sequence (STING-I3R; Supplementary Fig. S2A and S2B). Further investigation of this variant revealed an in-frame stop codon within the retained intron sequence, suggesting this variant may be a null allele. To verify whether this novel splice variant is present in primary AML, we cloned and sequenced *STING* from cDNA of primary bone marrow aspirates from 7 patients with AML. By this technique, 2 of 7 patients possess sequence-verified expression of this novel splice variant (Fig. 3A; Supplementary Fig. S2C). As validation, we performed PCR with primers designed to specifically amplify the STING-I3R allele, and with this approach observed the variant in 5 of 7 patient samples but not found in normal donors (Fig. 3C and D). In addition, 2 patients expressed an additional novel splice variant lacking *STING* exon 4 (STING-Ex4D; Fig. 3A; Supplementary Fig. S2D) that results in a frameshift. Together, these observations describe two previously unidentified alternatively spliced isoforms of STING found in human AML cell lines and primary bone marrow aspirates that may be functionally deficient.

### Alternatively Spliced STING Isoforms are Unresponsive to STING Agonists

**FIGURE 4:**
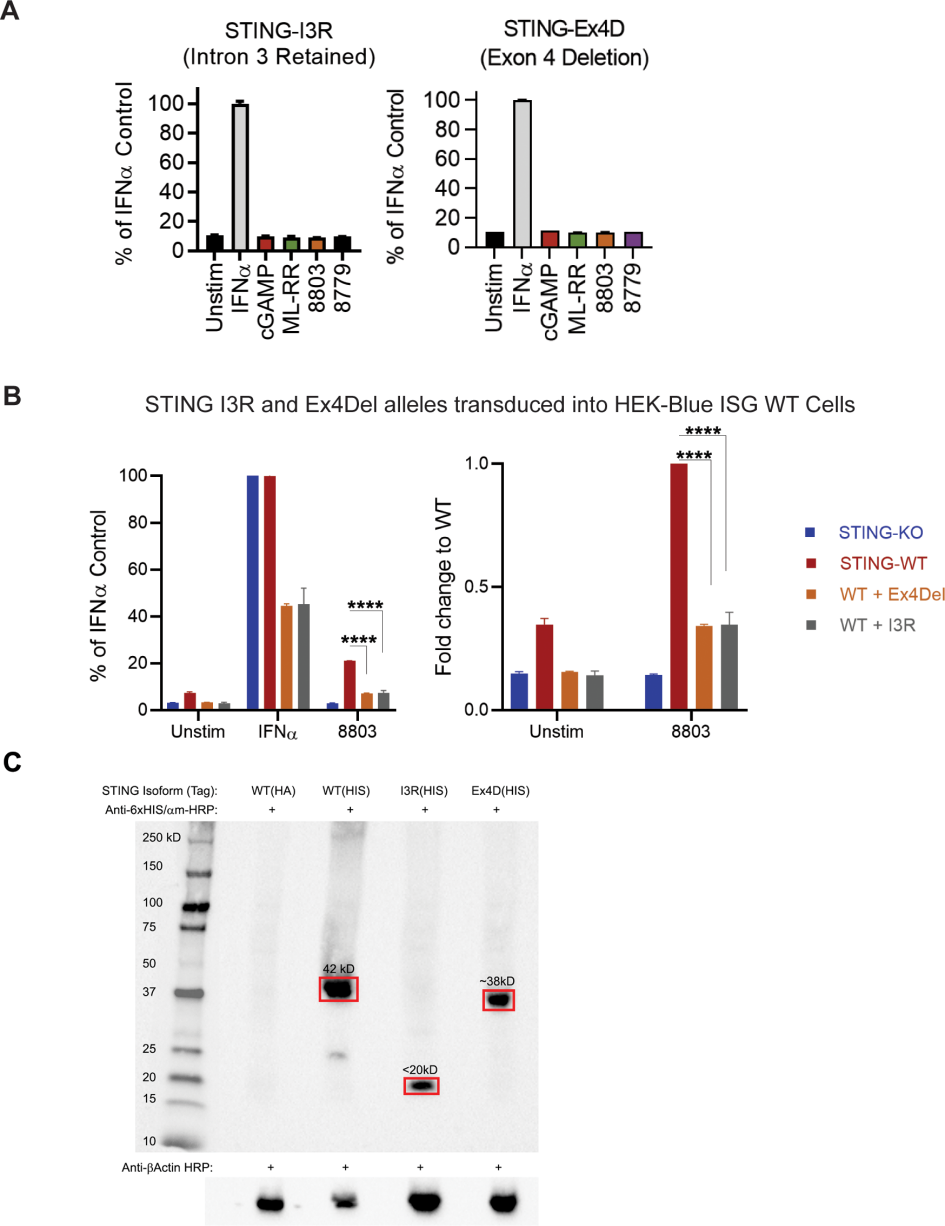
Novel STING alleles fail to respond to STING agonists. HEK-Blue-ISG-KO-STING cells were reconstituted with indicated novel STING variants (*n* = 2; **A**) or STING variants plus WT STING by retroviral transduction (**B**), then were exposed to 100 µg/mL indicated CDN for 24 hours (*n* = 2). IRF3/9 activity was measured by SEAP assay. IFNα positive control indicates presence and functionality of the IRF3/9 SEAP reporter in these cells. Statistical significance in B was calculated using repeated measures one-way ANOVA test. ns, not significant; *, *P* < 0.05; **, *P* < 0.01; ***, *P* < 0.001; ****, *P* < 0.0001. **C,** STING knockout 293 T cells were transduced to express human WT STING with an HA or 6xHIS tag, the STING-I3R isoform with a 6xHIS tag, or the STING-Ex4D isoform with a 6xHIS tag. Cell lysates were Western blotted with anti-6xHIS antibody (Invitrogen) followed by anti-mouse HRP (Cell Signaling Technology). Anti-human β-actin staining is shown as a loading control (Abcam).

To determine whether the *STING* splice variants identified above are functionally active or null alleles, we stably expressed them in STING-deficient HEK-Blue-ISG-KO-STING reporter cells and exposed them to our panel of potent CDNs. Successful reconstitution was verified by flow cytometric selection and PCR (Fig. 3C; Supplementary Fig. S3). HEK-Blue-ISG-KO-STING cells reconstituted with either novel splice variant failed to show any detectable STING activation, even at extremely high CDN concentrations of 100 µg/mL (Fig. 4A). Furthermore, we observed that these isoforms do not elicit any STING activation in HEK-Blue-ISG-STING WT cells, thereby suggesting they exert a dominant negative effect over WT STING (Fig. 4B). Finally, we confirmed that these novel isoforms are stably expressed at the protein level in 293 cells and have the predicted molecular weight via Western blot analysis (Fig. 4C). Therefore, we conclude that alternative splicing of *STING* can lead to expression of novel dysfunctional STING alleles in AML, which may contribute to the insensitivity of AML to CDN-mediated STING activation.

## Discussion

In this study, we investigated suppression of STING-mediated IFNβ production in AML. Using two novel CDNs, we confirmed that most AML cell lines are poor producers of IFNα/β protein following CDN exposure. We did not observe dysregulation of *STING* or *cGAS* expression or induction of exogenous regulators of CDN availability including *TREX1*, *TREX2*, or *ENPP1*. Rather, we found evidence of manipulation of the STING locus at genetic and transcriptional levels. Known germline STING isoforms including the HAQ and R232H alleles—present in less than a quarter of the human population individually (10)—were found in 5/8 and 3/8 of our tested AML lines, respectively. Given this striking enrichment compared with expected HAQ/R232H allele frequencies, it is tempting to speculate that WT STING may be protective against AML development, whereas disabling modifications of the STING locus can contribute to conditions in which AML development is supported. While a rigorous statistical evaluation of this hypothesis is beyond the scope of this study, we found biological evidence that supports it by identifying novel putative null STING isoforms in both cell lines and primary AML samples, which appear to arise due to alternative splicing. The first variant, STING-I3R, retains intron 3 in the coding sequence, introducing an in-frame stop codon to severely truncate STING. The second variant, STING-Ex4D, lacks exon 4 and creates a frameshift early in the STING coding sequence. We confirmed that these novel splice variants fail to respond to CDNs, and thus represent dysfunctional forms of STING. However, how these alleles exert a dominant negative effect in a heterozygous setting, with presence of the splice variant alleles over a WT STING background, remains unknown and requires further investigation. Potential mechanisms may involve these alleles disrupting normal protein folding or STING dimerization upon ligand binding to render downstream signaling dysfunctional.

Our results argue that AML may regulate STING signaling primarily through direct manipulation of the STING gene rather than altered expression of STING pathway components or exogenous regulators. A growing body of evidence supports the notion that *STING* can be subject to extensive genetic and transcriptional modulation. In addition to well-described single-nucleotide variants reported in the human population, there is evidence that alternative transcription start sites and mRNA splicing can produce alternate STING isoforms capable of functioning in a dominant-negative manner (11, 12). It remains to be determined which signals induce alternative splicing of *STING*, and whether these signals are specific to AML. Given that other components of innate immune signaling including MyD88, MAVS, RIG-I, and TLR4 can undergo alternative splicing in response to infection (22), it is intriguing to speculate that oncogenic signaling or cancer-related inflammation can initiate alternative splicing in STING and could be a generalizable phenomenon beyond the context of AML.

Our results do not, however, exclude additional mechanisms that can limit STING-mediated IFN production in AML. Differential CDN uptake efficiency may contribute to the observed differential responses of THP-1 and MV411 and overall insensitivity of other AML lines. However, it remains unclear by what means unformulated CDNs gain entry to the cytoplasm—whether through passive diffusion, pinocytosis, or receptor-mediated internalization. Therefore, it is difficult to propose a specific mechanism to support this hypothesis.

Notably, STING signaling has pleiotropic effects on myeloid cells in the tumor beyond production of type 1 IFNs, including but not limited to their effect on proliferation, proinflammatory repolarization, and metabolic modulation (23). In a prior study showing efficacy of STING agonists against murine syngeneic AML models, it was speculated that, in addition to the benefits of IFN produced by host cells, therapeutic benefit might also depend on conditioning of tumor vasculature by augmented levels of TNFα (24).

In summary, this study investigated potential mechanistic explanations for the poor induction of IFNα/β by human AML cells in response to CDN STING agonists. We did not observe downregulation of STING pathway components, or upregulation of enzymes that limit CDN availability at the RNA level. Rather, we identified two novel putative splice variants of the STING gene expressed in AML cell lines and primary bone marrow aspirates and confirmed that these spliced isoforms are nonfunctional. These observations suggest that regulation of the STING pathway in AML may primarily result from direct modulation of STING at the genetic and transcriptional level, rather than exogenous inhibition of the pathway.

## Supplementary Material

Supplementary Figure S1 IACS-8803 and -8779 are potent cyclic dinucleotide agonists of STING.

Supplementary Figure S2 Analysis of STING isoforms amplified from patient bone marrow samples.

Supplementary Figure S3 Representative gating scheme for sorting HEK cells transduced with STING variants.
