# Supplementary material for: Identification of Nonfunctional Alternatively Spliced Isoforms of STING in Human Acute Myeloid Leukemia
Source: Cancer Res Commun. 2024 Mar 25;4(3):911–8. doi: 10.1158/2767-9764.CRC-24-0095 (PMC10962316; doi:10.1158/2767-9764.CRC-24-0095)
Supplement: Supplementary Figure S1 — IACS-8803 and -8779 are potent cyclic dinucleotide agonists of STING. [file crc-24-0095-s01.pdf]

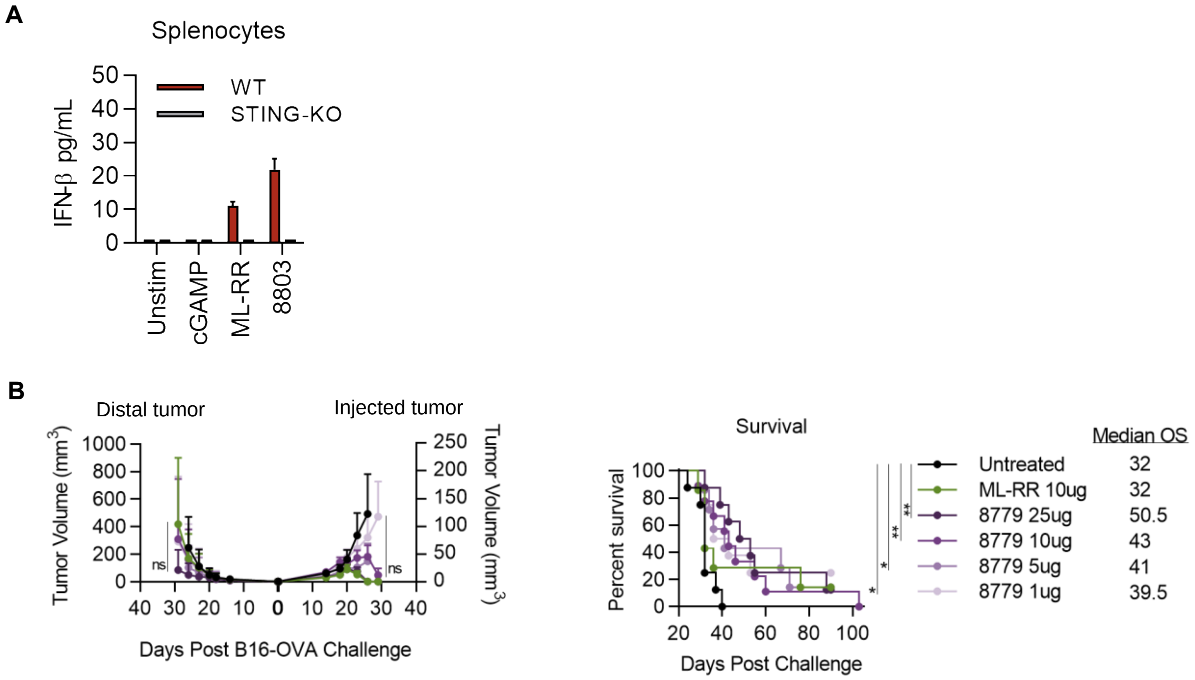

Supplementary Figure S1: Modified CDNs 8803 and 8879 activate STING. (A) Splenocytes derived from wild-type or STING-KO mice were treated with cGAMP, ML-RR, and 8803 and supernatants were analyzed for IFN-β by ELISA (n = 2). (B) Bilateral B16-OVA tumors treated with ML-RR or titrating doses of 8779 (n = 7-9). Statistical significance in (B) was calculated using unpaired t-test and Log-rank (Mantel-Cox) test. ns = not significant, * = p<0.05, ** = p<0.01, *** = p<0.001, **** = p<0.0001.
