# Supplementary material for: Identification of Nonfunctional Alternatively Spliced Isoforms of STING in Human Acute Myeloid Leukemia
Source: Cancer Res Commun. 2024 Mar 25;4(3):911–8. doi: 10.1158/2767-9764.CRC-24-0095 (PMC10962316; doi:10.1158/2767-9764.CRC-24-0095)
Supplement: Supplementary Figure S3 — Representative gating scheme for sorting HEK cells transduced with STING variants. [file crc-24-0095-s03.pdf]

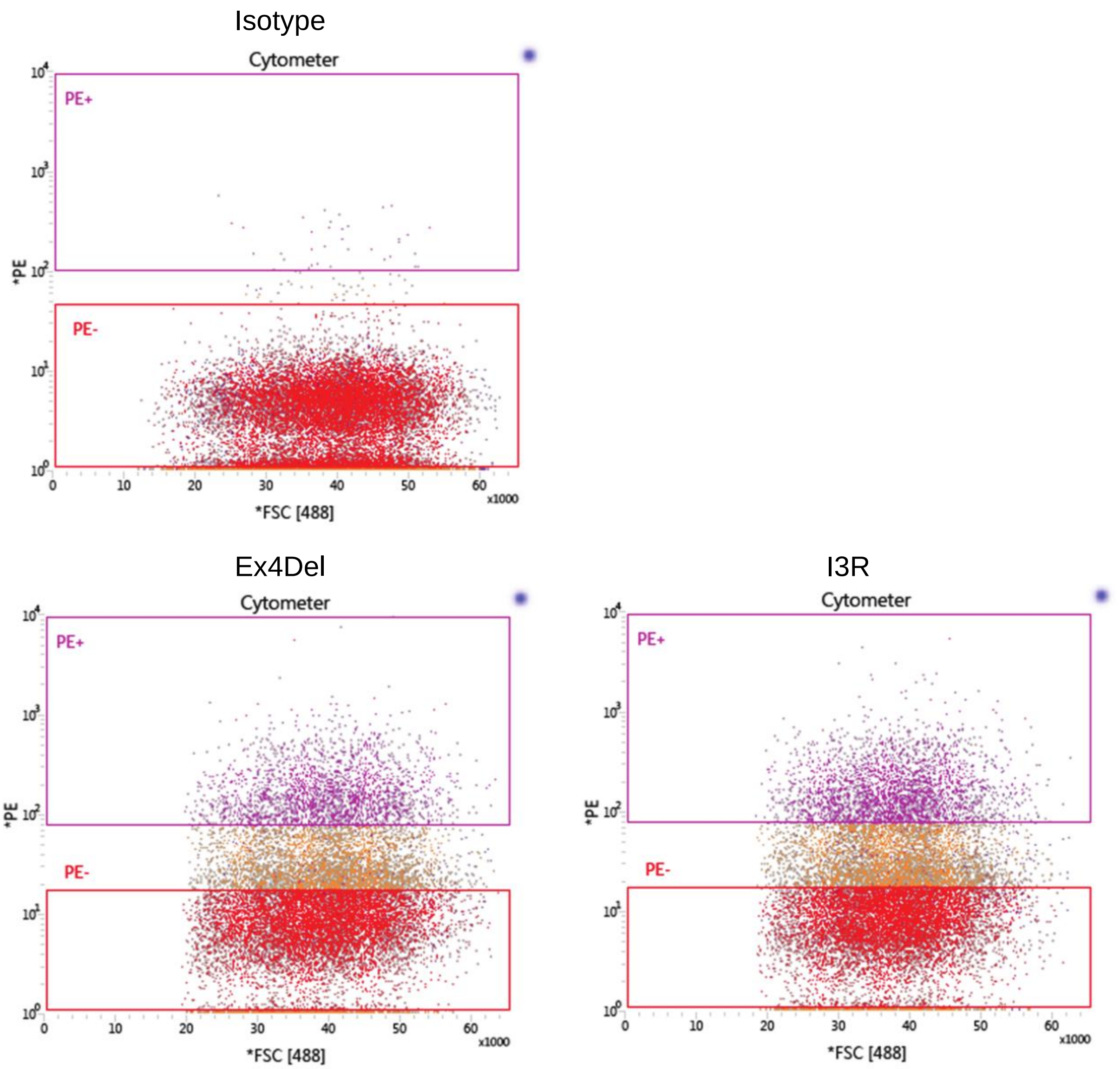

Supplementary Figure S3: Representative gating scheme for sorting HEK cells transduced with STING variants
