# Supplementary material for: Identification of Nonfunctional Alternatively Spliced Isoforms of STING in Human Acute Myeloid Leukemia
Source: Cancer Res Commun. 2024 Mar 25;4(3):911–8. doi: 10.1158/2767-9764.CRC-24-0095 (PMC10962316; doi:10.1158/2767-9764.CRC-24-0095)
Supplement: Supplementary Figure S2 — Analysis of STING isoforms amplified from patient bone marrow samples. [file crc-24-0095-s02.pdf]

A

	Patient 1	Patient 2	Patient 3	Patient 4	Patient 5	Patient 6	THP-1	MV411	MOLM13	U937	HL-60	OCI-AML3	Kasumi-1	AML193
Base 12 C -> T						X								
Base 36 T -> A		X												
Base 40 A -> C		X												
Base 41 G -> T		X												
Base 45 T -> A		X												
Base 144 C -> G	X	X			X		X	X	X	X	X	X	X	X
Base 300 A -> T												X		
Base 382 C -> T	X													x
Base 603 C -> T	X					X								
Base 899 C -> T	X													
Base 761 C -> T	X													
Base 899 C -> T														X
Base 977 C -> A														x
G230A		X					X		X	X	X	X		
R71H	X						X		X	X	X	X		
R232H	X							X					X	X
R293Q							X				X	X		
Intron 3 retain					X	X			X	X	X			
Exon 2 deletion	X													
Exon 4 deletion	X					X								
Exon 7 deletion		X	X		X	X	X		X	X	X	X		
Wildtype			X	X	X			X				X		X

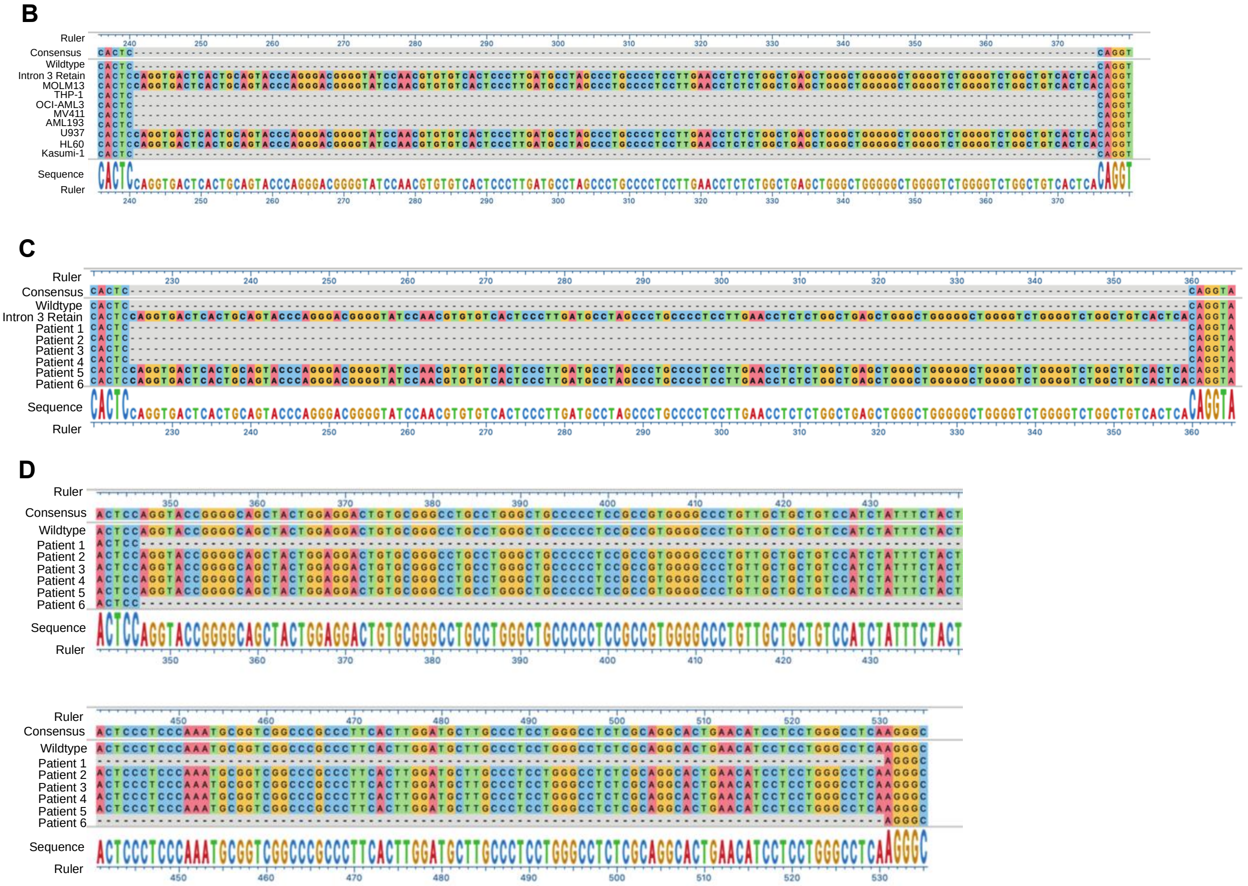

Supplementary Figure S2: Analysis of STING isoforms amplified from patient bone marrow samples. (A) Detected nucleotide mutations and STING variants in patient bone marrow samples. (B) Alignment of nucleotide sequences from human wild-type STING, intron 3 retained STING, and AML cell line derived sequences spanning intron 3. (C) Alignment of nucleotide sequences derived from patient samples spanning intron 3 and (D) exon 4.
